# Protocol Variations and Six-Minute Walk Test Performance in Stroke Survivors: A Systematic Review with Meta-Analysis

**DOI:** 10.1155/2015/484813

**Published:** 2015-01-20

**Authors:** A. Dunn, D. L. Marsden, E. Nugent, P. Van Vliet, N. J. Spratt, J. Attia, R. Callister

**Affiliations:** ^1^University of Newcastle, Callaghan, NSW 2308, Australia; ^2^Hunter Medical Research Institute, New Lambton Heights, NSW 2305, Australia; ^3^Hunter New England Local Health District, New Lambton Heights, NSW 2305, Australia

## Abstract

*Objective*. To investigate the use of the six-minute walk test (6MWT) for stroke survivors, including adherence to 6MWT protocol guidelines and distances achieved. *Methods*. A systematic search was conducted from inception to March 2014. Included studies reported a baseline (intervention studies) or first instance (observational studies) measure for the 6MWT performed by stroke survivors regardless of time after stroke.  *Results*. Of 127 studies (participants *n* = 6,012) that met the inclusion criteria, 64 were also suitable for meta-analysis. Only 25 studies made reference to the American Thoracic Society (ATS) standards for the 6MWT, and 28 reported using the protocol standard 30 m walkway. Thirty-nine studies modified the protocol walkway, while 60 studies did not specify the walkway used. On average, stroke survivors walked 284 ± 107 m during the 6MWT, which is substantially less than healthy age-matched individuals. The meta-analysis identified that changes to the ATS protocol walkway are associated with reductions in walking distances achieved. *Conclusion*. The 6MWT is now widely used in stroke studies. The distances achieved by stroke patients indicate substantially compromised walking ability. Variations to the standard 30 m walkway for the 6MWT are common and caution should be used when comparing the values achieved from studies using different walkway lengths.

## 1. Introduction

Compromised walking ability is a functional limitation significantly associated with poorer community integration following stroke [1] and improving walking capacity and endurance is often a key goal of stroke rehabilitation [2–5]. Functional walking tests, such as the 6MWT, were originally developed in the 1960s and used to assess people with cardiovascular and respiratory disease [6, 7]. More recently, the 6MWT has been used to characterise and monitor changes in walking capacity following stroke. The test is commonly used as a measure of walking endurance and is a significant predictor of community ambulation and integration in stroke survivors [8].

In 2002, the American Thoracic Society (ATS) published guidelines for the 6MWT [9] with the objective of standardising the protocol to encourage further application of the 6MWT and allow direct comparisons among different studies and populations. The ATS guidelines include test indications and contraindications, safety measures, and a step-by-step protocol and provide assistance with clinical interpretation. Key components of the protocol include the test location, walkway length, measurements, and instructions. According to the ATS protocol, the test should be performed on a flat, enclosed (indoor) walkway 30 m in length. This protocol requires 180° turns at either end of the walkway and additional space for turning is required. The guidelines advise that shorter walkway lengths require more directional changes and can reduce the distances achieved [9]. It is likely that the influence of directional changes may be amplified in the stroke population, who characteristically have impaired balance, asymmetrical gait patterns, and altered responses for turn preparation [8, 10, 11]. Conversely, reducing the number of directional changes may increase the distance achieved [12].

The aims of this systematic review were to synthesise the current literature that used the 6MWT and to investigate (1) the extent of its use in stroke survivors; (2) the characteristics of the stroke survivor populations studied; (3) the adherence to the ATS standard protocol; (4) the distances achieved; and (5) the influences of protocol modification and factors such as age, gender, disability, and time after stroke on distances achieved.

## 2. Methods

The conduct and reporting of this review was guided by the Preferred Reporting Items for Systematic Reviews and Meta-Analyses (PRISMA) statement and the Meta-Analysis of Observational Studies in Epidemiology (MOOSE) statement [13].

### 2.1. Search Strategy and Selection Criteria

A systematic computer-based search was undertaken of the databases MEDLINE, CINAHL, EMBASE, PsycINFO, AMED, SPORTDiscus, and COCHRANE from inception to 24th of March, 2014. The search strategy used for MEDLINE is outlined in Table 1 and was adapted to suit each database as required. Studies were deemed eligible if they were published in English, in peer-reviewed journals, and included the distance walked during the 6MWT by stroke survivors during the baseline (intervention studies) or first instance (observational studies). The World Health Organisation [14] defines stroke as “a focal (or at times global) neurological impairment of sudden onset, and lasting more than 24 hours (or leading to death), and of presumed vascular origin” and must have been diagnosed clinically. Both ischemic and haemorrhagic stroke were included at any time after stroke. Studies reporting data from mixed neurological groups that included people after stroke were excluded. Theses and articles published in abstract form only, including conference proceedings, were also excluded.

### 2.2. Selection of Studies

The author A. Dunn identified and obtained abstracts from the relevant studies based on title and classified each study as being a possible inclusion or definite exclusion according to the first inclusion criterion the study failed to meet. Studies were excluded if they were a subset from a larger study using the same participants, if the distance achieved on the 6MWT was not presented as text but rather a graph or chart, or if the 6MWT distance was not reported. Full-text versions of all possible inclusion studies were then retrieved and reviewed by the author A. Dunn who classified them as “include,” “exclude,” or “unsure.” This process was then independently conducted by the second reviewer E. Nugent. For instances where there was uncertainty or disagreement between authors, a consensus decision was reached through discussion and the involvement of a third reviewer, R. Callister, if necessary.

### 2.3. Data Extraction and Synthesis

Author A. Dunn then extracted the following variables from included studies:

- study characteristics: year published, participant numbers, and gender ratio;
- participant characteristics: age, gender, time since stroke, and disability score;
- 6MWT: distance achieved, protocol used, assistance provided, assistive devices used, and instructions given.

### 2.4. Quantitative Analysis of Adherence to ATS Protocol Guidelines

To date, there is no standardised approach to assessing the quality of reporting of observational studies such as adherence to protocol guidelines. As this is a systematic review looking only at baseline values rather than interventions, assessment of conventional methodological study quality is not applicable. Therefore, for the purpose of this review, a unique two-point scale was designed. Points were awarded as follows: one point for describing the protocol used and one point for referring to the ATS standards for the 6MWT. Therefore, a study could score zero, one, or two points unless it was published prior to the ATS standards (*n* = 3) in which case it could only achieve a score of zero or one.

### 2.5. Meta-Analysis

A meta-analysis was conducted to examine the influence of the 6MWT protocol variations, as well as age (continuous, years), gender (proportion of male participants in the study), physical disability score (converted to a continuous *z*-score), and time since stroke (continuous, months), on the distance walked. Studies were excluded from the meta-analysis if they did not perform the 6MWT indoors, on a flat walkway, with usual walking device, or if the walkway length was not described. Included studies were pooled into three groups based on the walkway length used: <30 m, =30 m, and >30 m shuttles. Studies where the test was performed on an oval or rectangular track were pooled together to create a fourth “continuous walkway” group. The ATS standards state 30 m or 100 ft walkway, which converts to 30.5 m. Any studies using a 30.5 m walkway were therefore included in the =30 m group. In studies that reported medians (IQRs), the medians were used as means and IQRs were converted to SDs by dividing the reported IQRs by 1.35; these approaches assume symmetrical distributions. A random-effects meta-regression was conducted where the square of each study's standard error was used as fixed values of the sampling variance. Statistical significance was accepted at the level of *α* ≤ 0.05. A second meta-regression was also conducted to examine the effects of age, gender, disability, and time since stroke in only those studies that used the 30 m walkway protocol.

## 3. Results

**Figure 1:**
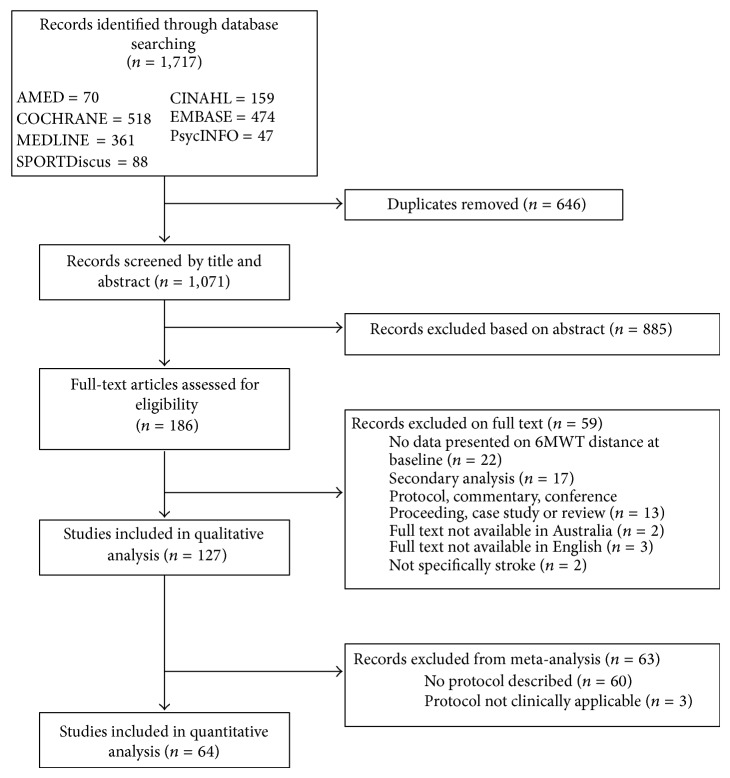
PRISMA flow diagram.

The search across seven databases yielded 1,717 citations from which 127 articles were identified for inclusion in the review.  Figure 1 details the flow of studies and reasons for exclusion throughout the selection process.  Table 2 summarises the study characteristics, participant characteristics, and 6MWT results. Of the included studies the first paper to use the 6MWT in stroke survivors was reported in 1998 [39]. The use of the test has since increased rapidly, with 11 papers published from 2000 to 2004, 48 papers published from 2005 to 2009, and 67 papers published from 2010 onward. Most (98%) papers were published since the publication of the ATS guidelines in 2002.

### 3.1. Participant Characteristics

The 127 studies reported on a collective sample size of 6,012 participants, including 3,654 males (61%), 2,188 females (36%), and 170 (3%) not specified. The participant eligibility criteria reported in studies included the following: participants greater than 6 months after stroke (*n* = 40 studies), no significant cognitive or communicative issues (*n* = 85 studies), mild or no cardiovascular or pulmonary problems (*n* = 66 studies), no orthopaedic or musculoskeletal problems or pain (*n* = 57 studies), no other neurological conditions (*n* = 42 studies), and participants able to meet a specified minimal or maximal walking speed either overground or on a treadmill (*n* = 17 studies). Only 77 studies reported being ambulant with or without assistance as eligibility criteria; however a further nine studies reported a minimal walking speed and four studies report ability to walk on a treadmill as inclusion criteria. These criteria imply the ability to walk, even if it is not explicitly stated.

Participant mean ages (SD) ranged from 45 (7) [78] to 76 (13) [70] years, and mean time since stroke varied from 11 (4) days [123] to 8.5 (0.9) years [36]. Thirteen studies did not specify time after stroke. Most studies included participants either within 6 months after stroke (*n* = 32), or 1–3 years (*n* = 27) or more than 3 years after stroke (*n* = 50), with much smaller numbers in the 6–12-month range (*n* = 9). A disability score was reported in only 58% of all studies. Of those that did report degree of disability, 12% reported the use of more than one scale. Common disability scales reported in these studies were the Fugl-Meyer Assessment (FM) (*n* = 24), Barthel Index (BI) (*n* = 13), Functional Independence Measure (FIM) (*n* = 12), Rivermead Mobility Index (*n* = 10), Functional Ambulatory Category (FAC) (*n* = 8), Chedoke McMaster (*n* = 6), Motor Assessment Scale (MAS) (*n* = 6), and the Motricity Index (MI) (*n* = 3). Other scales included the Walking Ability Questionnaire (*n* = 1), Dynamic Gait Index (DGI) (*n* = 1), the Late-Life Function and Disability Instrument (LLFDI) (*n* = 1), and the Modified Korean Barthel Index (*n* = 2). Subscale specific lower-limb measures were preferred in the meta-analysis, and these were available in the FM (motricity lower limb, lower extremity) (*n* = 10), FIM (mobility, walking capacity, and locomotion) (*n* = 7), Chedoke McMaster (leg) (*n* = 5), and MAS (walking) (*n* = 3) only.

### 3.2. Context of the 6MWT

A variety of terms were used to describe the purpose of the 6MWT, with common descriptors being a test of endurance (*n* = 26), capacity (*n* = 22), function (*n* = 21), performance (*n* = 6), and ability (*n* = 5). Twenty-four studies did not report the purpose of conducting the 6MWT in their study. On several occasions the test was performed in a different context or for a different purpose to that described in the ATS guidelines. The 6MWT was performed: to induce fatigue [124]; over a variety of obstacles such as foam mats and purpose built ramps [23]; and in nonstandard locations such as outdoors [30, 130, 138] including suburban streets or shopping centres [38]. Walking distances achieved in outdoor locations ranged from 175 ± 67 m to 463 ± 84 m [36], with participants walking 234 ± 66.5 m in the shopping centre [127]. Stroke survivors in two intervention groups who walked over foam mats and purpose built ramps [10] walked 102.6 ± 64.5 m and 78.5 ± 61.3 m.

### 3.3. Assistance and Instructions Provided

Assistance provided to participants during the 6MWT was reported in 24 studies, with most of these studies indicating no assistance or minor assistance required. The single point cane was the most commonly used assistive device (*n* = 426) used during the 6MWT. Other devices used included the walker (*n* = 106), quad cane (*n* = 77), and crutch (*n* = 5). A total of 251 stroke survivors used an ankle-foot orthosis (AFO) during the test. Reporting use of a “usual device” without specifying the device used was prevalent (*n* = 310 participants).

Only 44% of studies reported the instructions provided to participants for the 6MWT with variations evident between studies. The most common phrase used was to “cover as much distance as possible” (*n* = 20 studies), followed by “walk as far as you can” (*n* = 16). Five studies specified walking at a fast pace in their instructions, and eight studies instructed participants to walk at a comfortable speed. Encouragement was provided in 10 studies, and no encouragement or verbal feedback was given in 14 studies.

### 3.4. Quantitative Analysis of Adherence to ATS Protocol Guidelines

Including the three studies published prior to the ATS standards, 49 of the 127 studies received a zero score, indicating that they did not mention the ATS guidelines and did not describe a protocol for the test including walkway length or course design. Sixty-three studies received a score of one indicating that these studies either referenced the ATS standard or provided details on the walkway length used. Of these, 53 received one point for reporting the walkway length or course design, while only ten received one point for referencing the ATS guidelines. Only 15 studies scored two points, with only nine of these reporting a reference to the ATS guidelines and complying with them by using a 30 m walkway.

### 3.5. Modifications to the 6MWT Protocol Walkway Length

Only 25 of the 127 studies made a reference to the ATS guidelines for the 6MWT, with only nine of these clearly reporting the use of a 30 m walkway. Although referencing the ATS standards, six studies modified the protocol with variations including the use of a 25 m walkway [133], a 33 m walkway [127], a 50 m walkway [96, 128], a 100 m walkway [75], and a 30 m oval course [48]. Overall, 67 studies provided a description of the walkway whereas 60 studies did not provide any description of the length or shape of the walkway used. Of those providing a description, 27 reported using an indoor 30 m walkway in accordance with the guidelines while 10 used shorter walkway lengths, 14 used longer walkways lengths, and 14 used continuous walkways. Straight walkway lengths varied from 10 m [118] to 85 m [69–71].

### 3.6. Distances Achieved Using the 6MWT in Stroke Survivors

**Figure 2:**
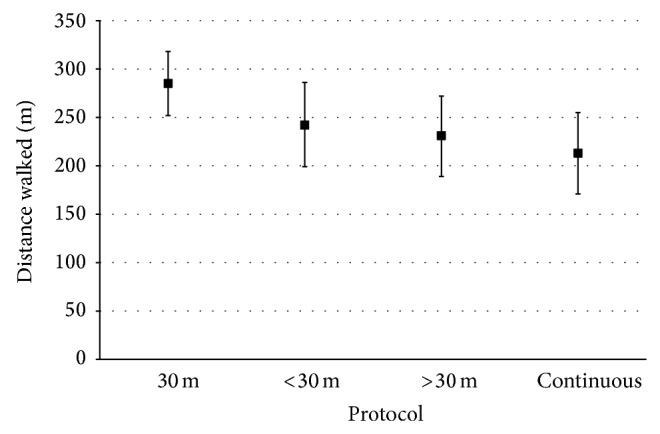
Distances achieved (point estimate, 95% CI) during the 6MWT based on walkway protocol used.

Sixty-four studies were included in the meta-analysis. The pooled distance walked across these 64 studies was 247.3 m (SE = 9.09). Heterogeneity was high with a tau (tau squared represents between study variance) of 84.9 m. We explored whether this heterogeneity was due to track type. Stroke survivors achieved a distance of 285 m (95% CI, 252–318 m) on a 30 m track. A significantly greater distance was achieved using the 30 m walkway compared to protocols with longer (231 m, 95% CI 189, 272, *P* = 0.048) or continuous (213 m, 95% CI 171, 255, *P* = 0.010) walkways (Figure 2); there was no significant difference between the 30 m walkway and shorter (242 m 95% CI 199, 286, *P* = 0.122) walkway lengths. Of the 60 studies that did not provide a description of the walkway used, an average distance of 246 ± 117 m was reported. Differences in distributions of age, gender, or time since stroke did not have a significant influence on distance walked (Table 3). Disability scores were only available on 74 (58%) studies and therefore could not be included in the analysis.

The regression analysis conducted in the 46 studies reporting use of the 30 m walkway found that none of the variables, that is, age (*P* = 0.479), gender (*P* = 0.768), or time after stroke (*P* = 0.909), were significantly associated with distance achieved on the 6MWT in stroke survivors (Table 4). Disability scores were only available on 12 (12/46, 26%) of these studies and therefore were not included in this analysis.

## 4. Discussion

This review has identified that the 6MWT is now in widespread use to assess aspects of walking-related performance in stroke survivor studies. Many of the study populations had to meet strict eligibility criteria including being able to meet a minimal walking speed and no comorbidities and therefore may only be selectively representative of stroke survivors. Many stroke survivors would not be able to meet the criteria for these studies, making a bias towards more well recovered, nondisabled, otherwise healthier participants. However, even in this selected population, the distances achieved on the 6MWT indicate substantial walking limitations in people after stroke. Overall, both the reporting of and adherence to the ATS guidelines for the 6MWT regarding the walkway length could be improved. Similarly, reporting of the instructions given prior to testing, as well as any assistance provided to participants during the test, requires attention in future publications. Alterations to the ATS protocol walkway, including shortening or extending the walkway length or using a continuous track, are more common than adhering to the 30 m walkway length. Consequently, a set of guidelines has been developed for future reporting of the 6MWT (Table 5). The findings from the meta-analysis were that the distance achieved during the 6MWT was associated with variations to the walkway length, but not in the manner one would predict. These findings have implications for the comparison of the values achieved using the 6MWT in different studies of stroke survivors.

The introduction of the ATS guidelines in 2002 was aimed at providing a protocol for consistency between studies. Of the 127 studies included in this review, 39 described a modified protocol whereas 60 studies did not specify the walkway length or walking course design. Of the 15 studies that received a reporting score of two, only nine reported both the ATS guidelines and used a 30 m walkway length in accordance with the guidelines: the remaining six studies referenced the ATS guidelines but reported using a modified track. Consequently, although the ATS guidelines may be referenced in a report, it cannot be assumed that there was adherence to the guidelines. Protocol modification was more common than compliance with the 30 m ATS standards, but no studies reported a reason for changing the protocol. Although there may be factors that necessitate or justify protocol changes, these reasons remain unreported. It is understandable that, in a setting where space is limited, there may be no other option than to use a walkway distance of less than 30 m. Reasons for lengthening the walkway are less clear. One explanation is that if space greater than 30 m in length is available, then a reason to extend the walkway length above the standard would be to decrease the effects of turning for stroke survivors in whom turning and balance ability may be compromised. We anticipated that the reduced turning requirements on the extended walkways and continuous tracks would result in longer distances being achieved than on the standard 30 m walkway. The results of the meta-analysis show the opposite, with these protocols resulting in shorter 6MWT distances. One possible explanation for these types of protocol changes would be to accommodate reduced turning abilities and more severe disability of participants in these studies, which may also explain the reduced walk distances achieved.

The main impact of varying the 6MWT track length or design is the extent of turning required throughout the test. Turning requires the integration of multiple sensory systems and utilises vestibular, visual, and proprioceptive information to appropriately move the body in space. Stroke survivors often experience difficulty during turning, possibly as a result of altered sensory, motor, and biomechanical systems [139]. This results in a differing orientation and sequencing of movements during turning compared to healthy controls [5], which requires more time to complete. Stroke survivors may take almost twice as many steps and twice as much time to complete a 180° turn compared to age-matched controls [140]. Similarly, two studies have reported significantly slower 180° turning times during the Timed Up and Go (TUG) in stroke [10, 141] with a similar finding by van Herk et al. (1998) [5] who demonstrated that the time taken to walk 10 m straight versus 5 m with return in stroke survivors was different, with the 5 m track with return requiring significantly more time to complete (*P* < 0.001). When comparing stroke survivors' performances on the 6MWT over 10, 20, and 30 m tracks, Ng et al. (2011) [8] quantified the increased number of turns associated with shortening the track and reported a significantly shorter distance achieved during the 10 m protocol, with the 30 m protocol reporting the longest distance walked. This is contradictory to the findings in this review; however when looking at percent difference there is some commonality. In the study by Ng et al., there is a reported reduction in distances achieved of 5% (20 m compared to 30 m) to 15% (10 m compared to 30 m). The results from the current meta-analysis suggest a reduction of 15% when comparing the <30 m protocol to the 30 m walkway length. Although this was not statistically significant, it is consistent with the magnitude of compromise found in the study by Ng et al. who measured the same population over multiple walkway lengths and could therefore make a direct comparison.

Another important finding from Ng et al. was that turning direction did not influence 6MWT distance independent of walkway length. The effects of turning direction on TUG times were also investigated in two other studies. Faria et al. [141] also found no difference in TUG times. In contrast Heung and Ng [142] found a significantly faster TUG time when turning towards the paretic side. There appears to be an expectation that turning towards the affected side would result in slower speeds and therefore decreased 6MWT distance; however as demonstrated in the literature, this may not be the case. Generally, the studies in this review did not specify the directions turned and therefore could not be analysed. It is noted however that in the clinical setting it is usually at the discretion of the individual performing the test as to which direction they turn during the 6MWT.

Our findings highlight the substantial effects of stroke on walking speed. Stroke survivors achieved an average distance of 285 m (95% CI 252, 318) on a standard 30 m track, whereas healthy older individuals >60 years achieve an average 6MWT distance of 499 m (95% CI 480, 519) [143]. The extent of the performance compromise on the 6MWT is striking, particularly when the studies in this review are largely reporting on a highly selected, high performing cohort of independently ambulant stroke survivors. When considering these distances achieved, it is important to also acknowledge any assistive devices used throughout the 6MWT. Overall, 948 stroke survivors used a walking aid, and 251 walked with an AFO. Allet et al. [16] found that stroke survivors walked approximately 15 m further during the 6MWT using a simple cane with an ergonomic handgrip than when walking with a 4-point cane or Nordic stick. This area requires further investigation and should be considered when interpreting data.

Of the variables age, gender, and time after stroke, none had a significant effect on the distances achieved. This is likely because meta-regression was performed using summary level data for each study, rather than individual patient data that would have more power to tease out heterogeneity. In healthy adults, the variables age and gender have been suggested as sources of variability on distances achieved [9].

Unfortunately, the effect of disability on distance walked could not be discerned in this review due to the large number of different disability scales used to describe stroke populations, the lack of consistency of scales between studies, and the underreporting of mobility related disability. An attempt to use an alternative measure of motor function, such as 10 m walking speed or balance, was also unsuccessful, as few of the studies in the meta-analysis reported on these measures.

The instructions provided to stroke survivors differ between studies, potentially impacting on the perceived goal of the 6MWT. In the current analysis, five studies reported wording to walk at a “fast” pace, while nine studies instructed participants to walk at a “comfortable” pace. According to the ATS guidelines, instructions should be informing the participant to walk “as far as possible for 6 minutes” with no mention of walking speed. The guidelines also provide standard encouragement wording to use. Of those studies that reported on the encouragement given throughout the test, 42% provided encouragement and 58% provided no verbal encouragement or feedback. The guidelines specify that the 6MWT should be performed indoors; however several studies reported using the 6MWT in different environments such as outdoors [36, 124, 126] including suburban streets and in shopping centres [127]. Although these trials were excluded from the meta-analysis, the exact implications of performing the 6MWT in different locations are unclear. Carvalho et al. [138] directly compared distanced achieved indoors and outdoors, concluding that stroke survivors in Group B (self-selected walking speed ≥0.8 m*·*s^−1^) achieved a greater distance in the outdoor setting, whereas for those in Group A (self-selected walking speed <0.8 m*·*s^−1^) there was no difference. It has also been suggested that gait parameters do not differ in stroke survivors when walking in different environments [127]. This is another factor that should be considered when interpreting the results reported from the 6MWT.

This review highlights the need for future researchers to be mindful in reporting their implementation of the 6MWT by describing the walkway used and rationales for deviations from the 30 m walkway or other aspects of the protocol. To assist future researchers we have developed a checklist of items for unambiguous reporting of the use of the 6MWT (Table 5). We acknowledge that changing the protocol may be necessary in some settings due to space restrictions. If the primary purpose of the test is to compare performances pre-post intervention then consistency is the main requirement between tests. Each setting may require their own documented protocol in order to maintain consistency between measurement times and test supervisors. The effects of level of disability on 6MWT performance could not be readily discerned due to the large number of different disability scales used, with many of these providing little indication of mobility impairment. Similarly, the extent of assistance provided, as well as the instructions given to the participants, requires attention in future studies. It can be concluded from the review that by changing the protocol researchers are limiting the ability to compare results between studies in stroke as well as other clinical populations. There is a lack of comprehensive reporting of the 6MWT protocol, which needs to be addressed in future research publications.

## Figures and Tables

**Table 1:** Search strategy used for MEDLINE.

1	Cerebrovascular Disorders.mp. or exp Cerebrovascular Disorders
2	Stroke.mp. or exp Stroke/
3	(cerebral or cerebellar or brainstem or vertebrobasilar).mp.
4	(infarct^*^ or ischemia or thrombo^*^ or embol^*^).mp.
5	3 and 4
6	(cerebral or brain or subarachnoid or intracerebral).mp.
7	(haemorrhage or haematoma or bleed^*^ or haemorrhage or hematoma).mp.
8	6 and 7
9	1 or 2 or 5 or 8
10	6MWT
11	Six minute walk^*^
12	6 minute walk^*^
13	10 or 11 or 12
14	9 and 13

**Table 2:** Description of included studies.

Study Year	Age (SD) years	Time since stroke (SD)	Number of participants (male : female)	Disability scale Score (SD)	Referenced ATS standards Protocol described Reporting score	Distance achieved (SD) meters
Ada et al. [15] 2003	I = 66 (11) C = 66 (11)	Months I = 28 (17) C = 26 (20)	27 19 : 08	SA-SIP I = 12.1 (5.5) C = 15.2 (5.2)	ATS = NR (0) Walkway = NR (0) Total = 0	I = 296 (96) C = 257 (113)

^*^Allet et al. [16] 2009	67.56 (9.5)	Days 41.84 (26.08)	25 NR	FIM total 100.84 (12.43)	ATS = No (0) Walkway = continuous circuit of 50 m circumference (1) Total = 1	Using 4 pt. cane = 101.4 (54.08) Using Nordic stick = 98.04 (51.28) Using simple cane = 115.48 (54.99)

^*^Batcho et al. [17] 2013	I = 57.97 (11.02) DO = 53.25 (10.53)	Months I = 37.7 (31.7) DO = 26.25 (10.69)	44 30 : 14	SIAS I = 56.5 (37–74)^x^ DO = 54.0 (37–73)^x^	ATS = No (0) Walkway = continuous square path 52 m in total (1) Total = 1	I = 243.6 (120.6) DO = 256.8 (123.5)

Bassile et al. [18] 2003	64.2^#^	Years 0.5–6	5 02 : 03	MAS 5.2	ATS = No (0) Walkway = NR (0) Total = 0	339.61 (155.82)

^*^Billinger et al. [19] 2012	61.2 (4.7)	Days 68.6 (40.1)	10 06 : 04	FM lower 27.4 (8.8)	ATS = Yes (1) Walkway = 100 ft (30.48 m) walkway (1) Total = 2	304.1 (167.5)

Blennerhassett and Dite [20] 2004	I1 = 56.3 (10.5) I2 = 53.9 (19.8)	Days I1 = 50.1 (49.2) I2 = 36.0 (25.1)	30 17 : 13	NR	ATS = No (0) Walkway = NR (0) Total = 0	I1 = 181 (85) I2 = 183 (85)

^*^Blennerhassett et al. [21] 2012	66 (49.3–72.0)^x^	NR	30 20 : 10	NR	ATS = No (0) Walkway = continuous rectangular track 86 m in total (1) Total = 1	312 (170–463)^x^

Bowden et al. [22] 2013	58.74 (12.97)	Months 22.70 (16.38)	27 19 : 08	FM 23.1 (4)	ATS = No (0) Walkway = NR (0) Total = 0	619.5 (290.8)

Brock et al. [23] 2011	I1 = 61.3 (13.0) I2 = 56.6 (15.8)	Days I1 = 60.3 (24.0) I2 = 63.6 (25.9)	26 19 : 07	NR	ATS = No (0) Walkway = 12.5 m long including a purpose built ramp and step and thin foam mats (1) Total = 1	I1 = 102.6 (64.5) I2 = 78.5 (61.3)

Brogårdh et al. [24] 2012	I = 61.3 (8.5) C = 63.9 (5.8)	Months I = 37.4 (31.8) C = 33.1 (29.2)	31 25 : 06	FIM I1 = 83.1 (3.1) I2 = 83.5 (3.2)	ATS = No (0) Walkway = NR (0) Total = 0	I = 305 (108) C = 393 (115)

Brogårdh et al. [25] 2012	64 (NR)	Months 42 (30)	50 41 : 09	NR	ATS = No (0) Walkway = NR (0) Total = 0	303 (130)

Byun et al. [26] 2011	58.9 (11.9)	Months 9.6 (4.5)	30 19 : 11	K-MBI 53.6 (24.1)	ATS = No (0) Walkway = NR (0) Total = 0	104 (96.4)

Carda et al. [27] 2011	I1 = 62.2 (11.7) I2 = 64.5 (12.5) I3 = 59.6 (14.3)	Months I1 = 46.9 (41.3) I2 = 52.3 (43.8) I3 = 43.9 (39.6)	69 34 : 35	FAC I1 = 3.9 (0.9) I2 = 4.0 (0.8) I3 = 3.8 (0.6)	ATS = No (0) Walkway = NR (0) Total = 0	I1 = 176.2 (98.5) I2 = 191.7 (98.6) I3 = 195.7 (81)

Carda et al. [28] 2012	63.9 (10.5)	Days 1,273 (1,460)	62 43 : 19	FAC 3 (2–4)^x^	ATS = No (0) Walkway = NR (0) Total = 0	Without HFO = 146.1 (99.7) With HFO = 169 (94.9)

Carroll et al. [29] 2012	72.4 (12.3)	Days 12.5 (6.25–34)^x^	50 21 : 29	BI 100 (90–100)^x^	ATS = Yes (1) Walkway = NR (0) Total = 1	158.6 (129.2)

^*^Carvalho et al. [30] 2008	60 (4.1)	Months 62 (33)	34 24 : 10	FM lower 30 (13–34)^x^	ATS = Yes (1) Walkway = indoors and outdoors over a 30 m course (1) Total = 2	In 365.2 (142.6) Out 373.6 (150.8)

^*^Carvalho et al. [31] 2013	59 (5.8)	Months 53 (36)	41 31 : 10	FM lower 28 (13–34)^x^	ATS = Yes (1) Walkway = 30 m walkway (1) Total = 2	372 (139)

^*^Chanruengvanich et al. [32] 2006	I = 62.83 (7.41) C = 63.18 (7.13)	NR	62 20 : 42	NR	ATS = No (0) Walkway = continuous rectangular path, total distance 30 m (1) Total = 1	I = 391.99 (82.6) C = 341.6 (89.97)

Da Silva et al. [33] 2013	54 (9)	Years 7.8^#^	12 06 : 06	FM 163.3^#^	ATS = Yes (1) Walkway = NR (0) Total = 1	274.2^#^

^*^Daly et al. [34] 2006	I = 57.7 (11.9) C = 63.6 (10.4)	Years I = 3.6 (3.8) C = 3.3 (2.1)	29 22 : 07	FM knee flex subscale I = 4 (2)^x^ C = 3 (2)^x^	ATS = No (0) Walkway = 30.5 m walkway (1) Total = 1	I = 186.6 (75.6) C = 128.3 (83.8)

^*^Daly et al. [35] 2011	I = 59 (NR) C = 62 (NR)	NR	44 32 : 12	FIM loco/mob I = 30 (2.75)^x^ C = 30 (8.50)^x^	ATS = No (0) Walkway = 30.5 m walkway (1) Total = 1	I = 530 (262.47) C = 416 (305.83)

^*^Danielsson et al. [36] 2011	59.7 (8.1)	Years 8.5 (0.9)	31 22 : 09	FM lower 29 (12)^x^	ATS = Yes (1) Walkway = 30 m walkway (1) Total = 2	352 (136)

^*^Dean et al. [37] 2001	62.7 (8.5)	NR	14 08 : 06	NR	ATS = No (published prior to ATS) Walkway = 50 m walkway (1) Total = 1	261.5 (128.4)

Donovan et al. [38] 2008	61.3 (11.1)	Months 46.5 (32.9)	30 21 : 09	NR	ATS = No (0) Walkway = 3 environments: clinic (150 m walkway), outdoors and in mall (1) Total = 1	Clinic = 244.4 (66.4) Street = 248.5 (77.4) Mall = 234.8 (66.5)

Duncan et al. [39] 1998	I = 67.3 (9.6) C = 67.8 (7.2)	Days I = 66 (NR) C = 56 (NR)	20 NR	FM lower I = 21.7 (NR) C = 23.2 (NR)	ATS = No (published prior to ATS) Walkway = NR (0) Total = 0	I = 149.66 (NR)^∧^ C = 169.47 (NR)^∧^

Duncan et al. [40] 2003	I = 68.5 (9) C = 70.2 (11.4) D = 74.6 (9.8)	Days I = 77.5 (28.7) C = 73.5 (27.1) D = 84.0 (27.2)	100 56 : 44	FM lower I = 24.1 (3.7) C = 23.7 (3.5) D = 26.0 (2.9)	ATS = No (0) Walkway = NR (0) Total = 0	I = 238.0 (103.9) C = 215.6 (94.8) D = 244.1 (88.6)

Duncan et al. [41] 2011	I1 = 60.1 (12.3) I2 = 63.3 (12.5) I3 = 62.6 (13.3)	Days I1 = 64.1 (8.3) I2 = 64.2 (9.0) I3 = 62.9 (8.0)	408 224 : 184	FM leg I1 = 23.7 (6.7) I2 = 24.8 (6.4) I3 = 24.7 (6.3)	ATS = No (0) Walkway = NR (0) Total = 0	I1 = 124.1 (77.5) I2 = 125.7 (81.8) I3 = 126.3 (75.0)

^*^Eng et al. [42] 2002	62.6 (8.5)	Years 4.4 (3.0)	25 17 : 08	CM lower 8.9 (2.4)	ATS = No (published prior to ATS) Walkway = continuous 42 m rectangular path (1) Total = 1	267.7 (89.7)

^*^Eng et al. [43] 2004	62.5 (8.6)	Years 3.5 (2.0)	12 11 : 01	CM lower 9.4 (2.5)	ATS = No (0) Walkway = continuous 42 m rectangular path (1) Total = 1	378.3 (123.1)

^*^Flansbjer et al. [4] 2005	M = 59 (7) F = 58 (5)	Months M = 16 (5) F = 18 (5)	50 38 : 12	NR	ATS = No (0) Walkway = 30 m walkway (1) Total = 1	T1 = 384 (132) T2 = 398 (136)

^*^Flansbjer et al. [44] 2008	I = 61 (5) C = 60 (5)	Months I = 18.9 (7.9) C = 20.0 (11.6)	24 14 : 10	NR	ATS = No (0) Walkway = 30 m walkway (1) Total = 1	I = 228 (137) C = 234 (134)

^*^Forsberg and Nilsagård [45] 2013	68.1 (11.2)	Years 4.6 (5.5)	67 42 : 25	RMI 37 (35–39)^x^	ATS = No (0) Walkway = 30 m walkway (1) Total = 1	247 (160–342)^x^

Fritz et al. [46] 2013	I = 67.6 (9.3) C = 64.5 (10.1)	Years I = 2.5 (2.6) C = 3.6 (3.2)	28 NR	FM I = 68.5 (21.7) C = 65.6 (18.0)	ATS = No (0) Walkway = NR (0) Total = 0	I = 285.4 (158.7) C = 263.2 (178.5)

^*^Fulk et al. [47] 2008	66.3 (14.3)	Days 33.7 (17.8)	37 20 : 17	FIM loco 5 (2–7)^x^	ATS = No (0) Walkway = 150 feet (45.72 m) at one site, 250 feet (76.20 m) at another two sites (1) Total = 1	T1 = 144.2 (136.3) T2 = 160.9 (146.3)

^*^Fulk et al. [48] 2010	65.7 (11.9)	Months 42.1 (36.1)	19 NR	FM lower 28.7 (5.7)	ATS = Yes (1) Walkway = continuous oval course 30 m in circumference (1) Total = 2	348.6 (144)

Geroin et al. [49] 2011	I1 = 63.6 (6.7) I2 = 63.3 (6.4) I3 = 61.1 (6.3)	Months I1 = 25.7 (6.0) I2 = 26.7 (5.1) I3 = 26.9 (5.8)	30 23 : 07	NR	ATS = No (0) Walkway = NR (0) Total = 0	I1 = 162.9 (52.1) I2 = 156.1 (62.9) I3 = 116.8 (75.2)

Gerrits et al. [50] 2009	54 (10)	Months 22 (18)	18 11 : 07	FAC 4.6 (SEM = 0.1)	ATS = No (0) Walkway = NR (0) Total = 0	186 (SEM = 25.9)

^*^Gjellesvik et al. [51] 2012	48.9 (10.6)	Years 7.2 (7.5)	8 04 : 04	NR	ATS = No (0) Walkway = 50 m walkway (1) Total = 1	474 (91)

Globas et al. [52] 2012	I = 68.6 (6.7) C = 68.7 (6.1)	Months I = 60.2 (46.6) C = 70.0 (67.4)	36 29 : 07	BI I = 91.7 (9.7) C = 88.3 (9.6)	ATS = No (0) Walkway = NR (0) Total = 0	I = 274.4 (113.0) C = 261.2 (177.0)

Gordon et al. [53] 2013	I = 63.4 (9.4) C = 64.9 (11.1)	Months I = 12.8 (3.6) C = 11.8 (3.6)	128 58 : 70	BI I = 94.3 (8.1) C = 91.5 (9.7)	ATS = No (0) Walkway = NR (0) Total = 0	I = 247.1 (141.5) C = 228.0 (138.7)

Hidler et al. [54] 2009	I = 59.9 (11.3) C = 54.6 (9.4)	Days I = 110.9 (62.5) C = 138.9 (60.9)	63 39 : 24	RMI I = 9.5 (0.5) C = 11.3 (0.6)	ATS = No (0) Walkway = NR (0) Total = 0	I = 118.2 (13.2)^∧^ C = 134.33 (14.1)^∧^

Hinson et al. [55] 2007	Bl = 62 (8) Wh = 66 (9)	Months Bl = 47 (57) Wh = 44 (54)	118 74 : 44	NR	ATS = No (0) Walkway = NR (0) Total = 0	Bl = 214 (108) Wh = 201 (123)

^*^Hoang et al. [56] 2012	64.6 (11.2)	Months 40 (42.2)	32 21 : 11	BI 89.7 (9.9)	ATS = No (0) Walkway = continuous 123 m circuit (1) Total = 1	201.8 (110.5)

Hornby et al. [57] 2008	I1 = 57 (10) I2 = 57 (11)	Months I1 = 50 (51) I2 = 73 (87)	48 30 : 18	NR	ATS = No (0) Walkway = NR (0) Total = 0	I1 = 170 (86) I2 = 170 (86)

Hwang et al. [58] 2013	1 = 54.6 (9.2) 2 = 54.9 (12.9)	Months 1 = 35.1 (18.8) 2 = 36.7 (19.0)	47 27 : 20	DGI 1 = 20.5 (2.7) 2 = 15.8 (4.8)	ATS = No (0) Walkway = NR (0) Total = 0	1 = 392.4 (68.9) 2 = 268.6 (74.5)

^*^Iosa et al. [59] 2012	62.7 (14.7)	Days 101 (36)	12 08 : 04	BI 70 (50–88)^x^	ATS = No (0) Walkway = 20 m walkway (1) Total = 1	226 (111)

Janssen et al. [60] 2008	I1 = 54.2 (10.7) I2 = 55.3 (10.4)	Months I1 = 12.3 (5.4) I2 = 18.3 (9.9)	12 06 : 06	FAC I1 = 4.5 (0.5) I2 = 4.7 (0.5)	ATS = No (0) Walkway = NR (0) Total = 0	I1 = 160.3 (134.4) I2 = 187.3 (92.0)

Jin et al. [61] 2012	I = 57 (6) C = 56 (7)	Months I = 18.5 (5.2) C = 17.9 (4.8)	133 94 : 39	RMI I = 10.3 (1.4) C = 10.2 (1.4)	ATS = No (0) Walkway = NR (0) Total = 0	I = 212.0 (63.5) C = 212.2 (50.1)

^*^Kang et al. [62] 2012	I1 = 55.9 (6.5) I2 = 56.3 (7.6) C = 56.1 (7.8)	Months I1 = 14.1 (4.4) I2 = 13.5 (4.0) C = 15.1 (7.4)	30 16 : 14	NR	ATS = No (0) Walkway = continuous 50 m track (1) Total = 1	I1 = 240.3 (20.9) I2 = 237.7 (25.4) C = 239.1 (22.0)

Kelley et al. [63] 2013	65.75 (9.48)	Years 2.87 (NR)	20 13 : 07	NR	ATS = No (0) Walkway = NR (0) Total = 0	51.61 (26.15)

^*^Kelly et al. [1] 2003	66 (48–73)^x^	Days 30 (19–39)^x^	17 13 : 04	NR	ATS = No (0) Walkway = 20 m walkway (1) Total = 1	301.8 (202.8–384.9)^x^

^*^Kim et al. [64] 2014	62.2 (11.7)	Days 32.6 (24.7)	55 37 : 18	KM-BI 74 (17.6)	ATS = No (0) Walkway = 50 m walkway (1) Total = 1	262.8 (120.7)

^*^Kluding and Gajewski [65] 2009	57.6 (11.0)	Months 45.4 (42.8)	26 14 : 12	NR	ATS = No (0) Walkway = 100 feet (30.48 m) walkway (1) Total = 1	202.4 (134.3)

Kluding et al. [66] 2011	63.7 (9.1)	Months 50.4 (37.9)	9 05 : 04	FM 87.7 (29.1)	ATS = No (0) Walkway = NR (0) Total = 0	760 (696.3)

Kuys et al. [67] 2011	I = 63 (14) C = 72 (17)	Days I = 52 (32) C = 49 (30)	30 12 : 18	Modified BI I = 76 (18) C = 80 (9)	ATS = No (0) Walkway = NR (0) Total = 0	I = 177 (130) C = 219 (147)

Lam et al. [68] 2010	66.8 (SEM = 1.1)	Months 59.0 (SEM = 9.28)	52 34 : 18	NR	ATS = No (0) Walkway = NR (0) Total = 0	253.25 (SEM = 19.38)

^*^Langhammer et al. [69] 2006	74 (NR)	NR	57 31 : 26	MAS walk 3.5 (2.5)	ATS = No (0) Walkway = 85 m walkway (1) Total = 1	234 (208)

^*^Langhammer et al. [70] 2008	I1 = 76.0 (12.7) I2 = 72.0 (13.6)	NR	75 43 : 32	BI I1 = 56.6 (38.9) I2 = 66.0 (39.0)	ATS = No (0) Walkway = 85 m walkway in hospital and different institutions including patients' homes or outdoors (1) Total = 1	I1 = 187.9 (211.1) I2 = 221.5 (197.8)

^*^Langhammer and Stanghelle [71] 2010	I1 = 74.0 (13.3) I2 = 75.0 (10.4)	Days I1 = 419 (1,034) I2 = 349 (820)	39 16 : 23	MAS i3 I1 = 5.4 (NR) I2 = 5.3 (NR)	ATS = No (0) Walkway = 85 m walkway (1) Total = 1	I1 = 277.7 (139.9) I2 = 299.4 (159.3)

Lee et al. [72] 2005	69 (11)	Months 43 (32)	11 09 : 02	NR	ATS = No (0) Walkway = NR (0) Total = 0	324.4 (173.1)

Lee et al. [73] 2008	I1 = 67.2 (10.6) I2 = 62.9 (9.3) I3 = 60.5 (10.6) C = 65.3 (6.0)	Months I1 = 52.4 (2.2) I2 = 44.2 (63.9) I3 = 63.2 (40.5) C = 65.8 (42.3)	48 28 : 20	NR	ATS = No (0) Walkway = NR (0) Total = 0	I1 = 249.3 (158.3) I2 = 239.8 (141.0) I3 = 266.0 (123.5) C = 273.2 (162.1)

Lord et al. [74] 2008	I1 = 64.2 (14.8) I2 = 60.7 (17.6)	Days I1 = 83.1 (29.8) I2 = 80.3 (33.0)	30 18 : 12	NR	ATS = No (0) Walkway = NR (0) Total = 0	I1 = 125.1 (71.0) I2 = 142.3 (89.4)

^*^MacKay-Lyons et al. [75] 2013	I1 = 61.5 (15.4) I2 = 59.0 (12.7)	Days I1 = 23.3 (5.7) I2 = 23.1 (4.4)	50 29 : 21	CM I1 = 4.8 (1.5) I2 = 4.9 (1.2)	ATS = Yes (1) Walkway = continuous 100 m walkway (1) Total = 2	I1 = 188.7 (82.3) I2 = 195.5 (77.7)

Macko et al. [76] 2005	I = 63 (10) C = 64 (8)	Months I = 35 (29) C = 39 (59)	61 43 : 18	RMI I = 11.3 (0.4) C = 11.7 (0.4)	ATS = No (0) Walkway = NR (0) Total = 0	I = 232.0 (22.3)^∧^ C = 258.5 (33.2)^∧^

Macko et al. [77] 2008	70 (1.7)	Months 56 (19)	20 09 : 11	BI 74.8 (4)	ATS = No (0) Walkway = NR (0) Total = 0	T1 = 116 (15) T2 = 113 (14)

^*^Maeda et al. [78] 2009	45 (7)	Months 19 (9)	18 15 : 03	NR	ATS = No (0) Walkway = 30 m walkway (1) Total = 1	AFO = 174 (77) W/O AFO = 140 (69)

^*^Marsden et al. [79] 2010	I = 70.0 (9.0) C = 73.1 (9.3)	Months I = 37.2 (26.7) C = 39.0 (23.6)	25 19 : 06	NR	ATS = No (0) Walkway = 20 m walkway (1) Total = 1	I = 301.6 (121.5) C = 349.1 (124.2)

Mayo et al. [80] 2013	I1 = 67.7 (14.4) I2 = 67.8 (12.3)	Days I1 = 265.4 (131.8) I2 = 252.0 (165.3)	87 60 : 27	NR	ATS = No (0) Walkway = NR (0) Total = 0	I1 = 323.4 (19.5) I2 = 321.6 (17.1)

Mehrholz et al. [81] 2006	54^#^	Weeks Range: 3–12	6 05 : 01	FAC 2 (0)	ATS = No (0) Walkway = NR (0) Total = 0	96.7 (33.4)

Mehrholz et al. [82] 2007	62.8 (10.2)	Days 30.6 (15.5)	55 40 : 15	RMI 2.51 (1.62)	ATS = No (0) Walkway = NR (0) Total = 0	15.9 (34.3)

^*^Michael et al. [83] 2009	71 (NR)	Years 7.5 (NR)	10 07 : 03	NR	ATS = No (0) Walkway = 100 feet (30.48 m) walkway (1) Total = 1	256 (33.5)^∧^

Miklitsch et al. [84] 2013	I = 58 (11) C = 57 (12)	Months I = 9.8 (19.1) C = 9.1 (20.8)	40 25 : 15	BI I = 55 (45–80)^x^ C = 53 (45–75)^x^	ATS = Yes (1) Walkway = NR (0) Total = 1	I = 261 (158) C = 200 (129)

^*^Moriello et al. [85] 2011	67 (12.3)	Months 3.9 (1.6)	63 43 : 20	NR	ATS = No (0) Walkway = 15 m walkway (1) Total = 1	277 (136)

Mudge et al. [86] 2009	I = 76 (39–89)^x^ C = 71 (44–86)^x^	Years I = 3.33 (0.6–13.3)^x^ C = 5.8 (0.5–18.7)^x^	58 32 : 26	RMI I = 14 (6.1–15)^x^ C = 13.5 (9–15)^x^	ATS = No (0) Walkway = NR (0) Total = 0	I = 263 (110) C = 201 (99)

Mudge and Stott [87] 2009	67.4 (12.5)	Months 66 (61)	49 29 : 20	RMI 13 (6–15)^x^	ATS = No (0) Walkway = NR (0) Total = 0	230 (121)

^*^Muren et al. [88] 2008	58 (9)	Months 60 (27)	30 17 : 13	NR	ATS = Yes (1) Walkway = 30 m walkway (1) Total = 2	353 (137)

Murtezani et al. [89] 2009	49.8 (17.4)	NR	44 26 : 18	BI 68 (20.5)	ATS = No (0) Walkway = NR (0) Total = 0	336.6 (82.2)

^*^Ng and Hui-Chan [90] 2005	61.1 (6.8)	Years 5.6 (3.3)	11 06 : 05	NR	ATS = No (0) Walkway = 33 m walkway (1) Total = 1	202.3 (88)

^*^Ng and Hui-Chan [91] 2009	I1 = 56.5 (8.2) I2 = 57.8 (7.3) I3 = 56.9 (8.6) C = 55.5 (8.0)	Years I1 = 4.9 (3.9) I2 = 4.7 (2.8) I3 = 4.3 (3.8) C = 5.0 (3.0)	109 85 : 24	NR	ATS = No (0) Walkway = 33 m walkway (1) Total = 1	I1 = 202.6 (81.8) I2 = 191.9 (89.4) I3 = 175.9 (81.9) C = 195.6 (75.9)

^*^Ng et al. [8] 2011	58.5 (6.1)	Years 7.0 (6.5)	26 13 : 13	NR	ATS = No (0) Walkway = performed on 10, 20, and 30 m walkways turning towards affected and nonaffected sides separately (1) Total = 1	1 = 227.32 (79.07) 2 = 252.2 (95.31) 3 = 265.47 (94.16) 4 = 228.8 (84.20) 5 = 255.79 (92.14) 6 = 269.35 (107.75)

^*^Ng and Hui-Chan [92] 2012	57.4 (7.8)	Years 5.2 (3.7)	62 51 : 11	NR	ATS = No (0) Walkway = 33 m walkway (1) Total = 1	183.7 (84.4)

Nolan et al. [93] 2009	53.44 (11.50)	Months 54.89 (36.98)	18 14 : 04	NR	ATS = No (0) Walkway = NR (0) Total = 0	With AFO = 228.54 (103.93) Without AFO = 197.49 (104.13)

^*^Olawale et al. [94] 2011	I1 = 56.8 (6.4) I2 = 56.8 (8.3) C = 57.2 (5.9)	Months I1 = 10.2 (6.9) I2 = 10.7 (6.8) C = 10.3 (5.9)	60 34 : 26	NR	ATS = No (0) Walkway = 15 × 10 m rectangular course (1) Total = 1	I1 = 111.43 (50.96) I2 = 116.31 (49.39) C = 138.85 (65.33)

Ouellette et al. [95] 2004	I = 65.8 (2.5) C = 66.1 (2.1)	Months I = 31.8 (3.3) C = 25.6 (4.0)	42 28 : 14	LLFDI I = 52.7 (2.3) C = 55.6 (2.2)	ATS = No (0) Walkway = NR (0) Total = 0	I = 217.1 (30.5) C = 221.0 (34.0)

^*^Outermans et al. [96] 2010	I1 = 56.8 (8.6) I2 = 56.3 (8.6)	Days I1 = 22.5 (8.2) I2 = 23.5 (7.8)	43 36 : 07	NR	ATS = Yes (1) Walkway = 50 m walkway (1) Total = 2	I = 459.8 (145.8) I2 = 401.0 (131.5)

^*^Ovando et al. [97] 2011	53 (17)	Months 18 (11)	8 06 : 02	FM 25 (4.5)	ATS = No (0) Walkway = 30 m walkway (1) Total = 1	400.9 (136.0)

Pang et al. [98] 2005	I = 65.8 (9.1) C = 64.7 (8.4)	Years I = 5.2 (5.0) C = 5.1 (3.6)	63 37 : 26	NR	ATS = Yes (1) Walkway = NR (0) Total = 1	I = 328.1 (143.5) C = 304.1 (123.8)

^*^Park et al. [99] 2011	I = 59.38 (8.46) C = 56.92 (7.79)	Months I = 28.08 (12.59) C = 28.67 (17.96)	25 12 : 13	WAQ I = 46.38 (10.38) C = 48.75 (10.15)	ATS = No (0) Walkway = 20 m walkway (1) Total = 1	I = 166.23 (58.29) C = 151.83 (69.95)

^*^Patterson et al. [100] 2007	64 (10)	Months 48 (59)	74 43 : 31	NR	ATS = No (0) Walkway = 30.5 m walkway (1) Total = 1	216 (120)

^*^Patterson et al. [101] 2008	64 (8)	Months 20.55 (64.0)	39 25 : 14	NR	ATS = No (0) Walkway = 30.5 m walkway (1) Total = 1	227 (105)

^*^Peurala et al. [102] 2005	I1 = 53.3 (8.9) I2 = 51.2 (7.9) I3 = 52.3 (6.8)	Years I1 = 2.6 (2.4) I2 = 2.4 (2.6) I3 = 4.0 (5.8)	45 37 : 08	FIM I1 = 99.2 (12.8) I2 = 106.9 (10) I3 = 100.7 (11.4)	ATS = No (0) Walkway = 54 m walkway (1) Total = 1	I1 = 127.1 (87.2) I2 = 152.3 (89.6) I3 = 111.8 (57.3)

^*^Pohl et al. [12] 2002	72.1 (10.2)	Days 73.3 (26.8)	72 40 : 32	FM lower 24 (3.8)	ATS = No (0) Walkway = 100 feet (30.48 m) walkway (1) Total = 1	215.8 (91.6)

Polese et al. [103] 2013	56.4 (12.5)	Months 64.8 (53.6)	98 54 : 44	NR	ATS = Yes (1) Walkway = NR (0) Total = 1	356.6 (132.2)

^*^Pradon et al. [104] 2013	53.3 (13.7)	Months 16 (8)	24 12 : 12	NR	ATS = No (0) Walkway = 30 m walkway (1) Total = 1	273.8 (173.4)

Rabadi et al. [105] 2008	I1 = 75.00 (10.58) I2 = 73.58 (13.02)	Days I1 = 16.36 (15.70) I2 = 14.10 (11.23)	116 68 : 48	FIM motor I1 = 25.93 (12.41) I2 = 25.93 (11.78)	ATS = No (0) Walkway = NR (0) Total = 0	I1 = 26.69 (48.77)^∧^ I2 = 29.16 (38.56)^∧^

Rand et al. [106] 2009	66.5 (9.6)	Years 2.9 (2.4)	40 13 : 27	NR	ATS = Yes (1) Walkway = NR (0) Total = 1	318.8 (78.6)

Ryan et al. [107] 2011	63 (1)	Months 39 (7)	70 39 : 31	NR	ATS = No (0) Walkway = NR (0) Total = 0	633 (46)

Ryan et al. [108] 2011	I = 62.8 (10.3) C = 60.7 (12.8)	Days I = 21.5 (8.7) C = 19.7 (16.8)	44 35 : 09	CM I = 4.2 (1.1) C = 4.1 (1)	ATS = No (0) Walkway = NR (0) Total = 0	I = 190 (176) C = 270 (236)

^*^Salbach et al. [109] 2004	I1 = 71 (12) I2 = 73 (8)	Days I1 = 239 (83) I2 = 217 (73)	91 56 : 35	NR	ATS = No (0) Walkway = 20 m walkway (1) Total = 1	I1 = 209 (126) I2 = 204 (131)

Salbach et al. [110] 2014	71.1 (9.7)	Years 2.0 (1.1)	16 14 : 02	CM leg 5 (1.4)	ATS = Yes (1) Walkway = NR (0) Total = 1	254.9 (180.9)

^*^Schmid et al. [111] 2012	64.06 (8.78)	Months 52.00 (42.14)	77 58 : 19	NR	ATS = No (0) Walkway = 30 m walkway (1) Total = 1	269.75 (131.06)^∧^

Severinsen et al. [112] 2011	68 (9)	Months 18 (6)	48 35 : 13	FM 68 (25)	ATS = Yes (1) Walkway = NR (0) Total = 1	291 (171)

^*^Sibley et al. [113] 2008	68.1 (12)	Days 52.4 (13.6)	26 16 : 10	FIM 107.1 (10.2)	ATS = Yes (1) Walkway = 30 m walkway (1) Total = 2	343.6 (107.3)

Simpson et al. [114] 2011	67.6 (9.9)	NR	80 58 : 22	NR	ATS = Yes (1) Walkway = NR (0) Total = 1	275.9 (141.8)

^*^Singh et al. [115] 2013	I = 65.4 (9.8) C = 67.0 (8.4)	Months I = 40.5 (41.8) C = 34.9 (23.6)	28 16 : 12	BI I = 87.00 (15.45) C = 92.31 (12.69)	ATS = No (0) Walkway = 30 m walkway (1) Total = 1	I = 162.40 (78.97) C = 209.92 (176.53)

Stookey et al. [116] 2013	61.2 (8.4)	NR	23 11 : 12	NR	ATS = No (0) Walkway = NR (0) Total = 0	T1 = 278 (123.2) T2 = 307.7 (142.0) T3 = 305.1 (138.3)

^*^Stookey et al. [117] 2014	61.5 (9.8)	NR	43 30 : 13	NR	ATS = No (0) Walkway = 100 ft (30.48 m) walkway (1) Total = 1	242 (115)

^*^Stuart et al. [118] 2009	I = 66.8 (1.4) C = 70.0 (1.7)	Years I = 4.2 (0.8) C = 3.5 (0.5)	78 54 : 24	BI I = 79.5 (2.6) C = 85.4 (2.0)	ATS = No (0) Walkway = 10 m walkway (1) Total = 1	I = 184.0 (11.8) C = 194.4 (9.2)

^*^Sullivan et al. [119] 2007	I1 = 60.6 (13.7) I2 = 63.4 (8.6) I3 = 58.2 (15.2) I4 = 61.4 (11.2)	Months I1 = 27.5 (16.1) I2 = 28.4 (19.0) I3 = 23.1 (15.0) I4 = 20.7 (14.4)	80 45 : 35	FM lower I1 = 24.5 (5.5) I2 = 24.4 (4.5) I3 = 24.2 (4.0) I4 = 22.1 (6.3)	ATS = No (0) Walkway = continuous, 18 m oval walkway (1) Total = 1	I1 = 189.3 (99.9) I2 = 170.0 (115.2) I3 = 187.6 (99.9) I4 = 190.0 (135.4)

^*^Tang et al. [120] 2006	64.6 (14.4)	Days 50.3 (17)	36 14 : 22	CM 5.1 (1)	ATS = Yes (1) Walkway = 30 m walkway (1) Total = 2	341.6 (107.9)

^*^Tang et al. [121] 2009	I = 64.7 (SEM = 3.6) C = 65.7 (SEM = 2.3)	Days I = 19.1 (SEM = 3.8) C = 14.9 (SEM = 2.3)	36 NR	FIM I = 84.0 (SEM = 4.1) C = 83.9 (SEM = 4.2)	ATS = Yes (1) Walkway = 30 m walkway (1) Total = 2	I = 207.0 (46.6) C = 198.9 (40.2)

Tanne et al. [122] 2008	I = 61 (10) C = 58 (5)	Days I = 65 (37) C = 93 (60)	48 43 : 05	FIM I = 123 (5) C = 122 (5)	ATS = No (0) Walkway = NR (0) Total = 0	I = 444 (90) C = 438 (101)

Toledano-Zarhi et al. [123] 2011	I = 65 (10) C = 65 (12)	Days I = 11 (5) C = 11 (4)	28 21 : 07	NR	ATS = No (0) Walkway = NR (0) Total = 0	I = 415.9 (172.5) C = 459.3 (116.3)

^*^Tseng and Kluding [124] 2009	56.8 (11.8)	Months 47.6 (51.2)	9 02 : 07	FM 79.4 (32.8)	ATS = No (0) Walkway = 100 feet (30.48 m) walkway (1) Total = 1	295.5 (171.4)

^*^van Bloemendaal et al. [125] 2012	58.8 (9.8)	Months 24.7 (25.3)	75 47 : 28	NR	ATS = Yes (1) Walkway = 30 m walkway (1) Total = 2	472.5 (156.1)

van de Port et al. [126] 2012	I = 56 (10) C = 58 (10)	Days I = 91 (42) C = 103 (51)	250 162 : 88	RMI I = 12.67 (1.58) C = 12.35 (2.00)	ATS = No (0) Walkway = NR (0) Total = 0	I = 339 (120) C = 306 (135)

^*^Verheijde et al. [127] 2013	70 (13)	Days 52 (87)	43 33 : 10	LEFS 33 (18)	ATS = Yes (1) Walkway = 33 m walkway (1) Total = 2	240 (130)

^*^Verma et al. [128] 2011	I = 53.27 (8.53) C = 55.07 (6.80)	Weeks 6.3 (3.2)	30 22 : 08	BI I = 60 (9.82) C = 53 (16.98)	ATS = Yes (1) Walkway = 50 m walkway (1) Total = 2	I = 115.2 (43.30) C = 96.67 (64.23)

^*^Westlake and Patten [129] 2009	I1 = 58.6 (16.9) I2 = 55.1 (13.6)	Months I1 = 43.8 (26.8) I2 = 36.8 (20.3)	16 13 : 03	FM lower I1 = 23.0 (4.3) I2 = 21.4 (5.1)	ATS = No (0) Walkway = 39 m walkway (1) Total = 1	I1 = 267.3 (187.2) I2 = 234.3 (141.2)

Wevers et al. [130] 2011	60.7 (10.9)	Days 266 (38)	27 21 : 06	NR	ATS = No (0) Walkway = outdoor 30 m walkway (1) Total = 1	1 = 408 (132) 2 = 417 (139) 3 = 413 (127) 4 = 422 (132)

White et al. [131] 2013	65.76 (11.01)	Years 2.11 (1.76)	21 16 : 05	NR	ATS = No (0) Walkway = NR (0) Total = 0	377.82 (176.19)

Wing et al. [132] 2008	60.2 (14.1)	Months 40.9 (29.1)	35 21 : 14	FM motor 31.8 (SEM = 3.2)	ATS = No (0) Walkway = NR (0) Total = 0	228.6 (SEM = 22.3)

^*^Yang et al. [133] 2006	I = 56.8 (10.2) C = 60.0 (10.4)	Months I = 62.7 (27.4) C = 64.0 (40.4)	48 32 : 16	NR	ATS = Yes (1) Walkway = 25 m walkway (1) Total = 2	I = 352.3 (71.7) C = 335.0 (79.6)

^*^Yang et al. [134] 2014	I1 = 53.9 (10.5) I2 = 54.5 (8.0)	Months I1 = 11.1 (8.1) I2 = 11.1 (9.7)	30 22 : 08	BI I1 = 17.4 (2.2) I2 = 16.5 (3.8)	ATS = No (0) Walkway = 10 m walkway (1) Total = 1	I1 = 216.4 (107.4) I2 = 193.1 (127.3)

Yiu et al. [135] 2012	67.41 (10.13)	Days 96.9 (69.0)	98 71 : 27	NR	ATS = No (0) Walkway = NR (0) Total = 0	267.65 (139.53)

^*^Zalewski and Dvorak [136] 2011	71.3 (9.5)	NR	17 14 : 03	NR	ATS = No (0) Walkway = continuous 200 m track (1) Total = 1	258.5 (146.0)

Zedlitz et al. [137] 2012	I1 = 54.8 (9.1) I2 = 55.6 (8.8)	Years I1 = 4.4 (4.2) I2 = 3.3 (3.9)	83 43 : 40	MI I1 = 90.2 (15.0) I2 = 90.1 (12.1)	ATS = Yes (1) Walkway = NR (0) Total = 1	I1 = 438 (123) I2 = 437 (107)

^*^Included in meta-analysis; ^∧^converted from feet to meters; ^#^calculated from individual data; ^x^median (IQR); AFO = ankle-foot orthoses; ATS = American Thoracic Society; BI = Barthel Index; C = control; CM = Chedoke-McMaster; DGI = Dynamic Gait Index; DO = Drop Out group; FAC = Functional Ambulation Category; FIM = Functional Independence Measure; FM = Fugl-Meyer; HFO = hip flexor orthoses; I = intervention; K-MBI = Korean Modified-Barthel Index; LEFS = Lower Extremity Functional Scale; LLFDI = Late-Life Function and Disability Instrument; MAS = Motor Assessment Scale; MI = Motricity Index; NR = not reported; SA-SIP = Stroke Adapted Sickness Impact Profile; SEM = Standard Error of the Mean; SIAS = Stroke Impairment Assessment Set; WAQ = Walking Ability Questionnaire.

**Table 3:** Meta-regression coefficients for all studies.

Effect	Estimate	Std. Err.	DF	*t* value	Probt	Lower CI	Upper CI
Age	0.6	1.7	75	0.38	0.708	−2.7	4.0
Proportion males	0.6	0.8	75	0.75	0.455	−1.0	2.2
Time since stroke	0.5	0.4	75	1.16	0.251	−0.3	1.3

**Table 4:** Meta-regression coefficients for 30 m protocol subgroup.

Effect	Estimate	Std. Err.	DF	*t* value	Probt	Lower CI	Upper CI
Age	−2.4	3.3	25	−0.72	0.479	−9.1	4.4
Proportion males	0.4	1.4	25	0.30	0.768	−2.5	3.3
Time since stroke	−0.1	0.7	25	−0.12	0.909	−1.5	1.4

**Table 5:** Checklist for reporting of the 6MWT.

Checklist for reporting of the 6MWT	Check
(1)	
(a) Acknowledge awareness of the ATS standards by referencing	□
(b) Report walkway length even if it is 30 m	□
(c) If deviating from ATS standards, describe changes (walkway length, course layout, and location) and explain reason	□

(2)	
(a) Describe the instructions given prior to the test	□
(b) Describe any encouragement provided during the test	□

(3) Report the number and type of assistive devices used	□

(4) Report any assistance or support provided to participants	□

(5)	
(a) Report the demographics of the population including disability level	□
(b) Report clearly the inclusion and exclusion criteria	
